# Uptake Mechanism of ApoE-Modified Nanoparticles on Brain Capillary Endothelial Cells as a Blood-Brain Barrier Model

**DOI:** 10.1371/journal.pone.0032568

**Published:** 2012-03-01

**Authors:** Sylvia Wagner, Anja Zensi, Sascha L. Wien, Sabrina E. Tschickardt, Wladislaw Maier, Tikva Vogel, Franz Worek, Claus U. Pietrzik, Jörg Kreuter, Hagen von Briesen

**Affiliations:** 1 Department of Cell Biology and Applied Virology, Fraunhofer Institute for Biomedical Engineering, St. Ingbert, Germany; 2 Institute of Pharmaceutical Technology, Goethe-University, Frankfurt am Main, Germany; 3 Institute of Pathobiochemistry, University Medical Center of the Johannes Gutenberg-University Mainz, Mainz, Germany; 4 Laboratory of Pathology and Radiation Biology Branch, National Cancer Institute, National Institute of Health, Bethesda, Maryland, United States of America; 5 Bundeswehr Institute of Pharmacology und Toxicology, München, Germany; Biological Research Center of the Hungarian Academy of Sciences, Hungary

## Abstract

**Background:**

The blood-brain barrier (BBB) represents an insurmountable obstacle for most drugs thus obstructing an effective treatment of many brain diseases. One solution for overcoming this barrier is a transport by binding of these drugs to surface-modified nanoparticles. Especially apolipoprotein E (ApoE) appears to play a major role in the nanoparticle-mediated drug transport across the BBB. However, at present the underlying mechanism is incompletely understood.

**Methodology/Principal Findings:**

In this study, the uptake of the ApoE-modified nanoparticles into the brain capillary endothelial cells was investigated to differentiate between active and passive uptake mechanism by flow cytometry and confocal laser scanning microscopy. Furthermore, different *in vitro* co-incubation experiments were performed with competing ligands of the respective receptor.

**Conclusions/Significance:**

This study confirms an active endocytotic uptake mechanism and shows the involvement of low density lipoprotein receptor family members, notably the low density lipoprotein receptor related protein, on the uptake of the ApoE-modified nanoparticles into the brain capillary endothelial cells. This knowledge of the uptake mechanism of ApoE-modified nanoparticles enables future developments to rationally create very specific and effective carriers to overcome the blood-brain barrier.

## Introduction

The blood-brain barrier (BBB) is one of the most important and impermeable physiological barriers in the organism. Its discovery in 1885 goes back to Paul Ehrlich who showed in animal experiments that after intravenous injection of trypan blue all tissues with the exception of the brain and the spinal cord were colored. Finally, after development of special electron microscopic methods in the 60's, the cerebral endothelial cells could be identified as the cellular basis of the blood-brain barrier. These brain capillary endothelial cells clearly differ from the endothelial cells in the remaining body in both morphological and metabolic properties. The endothelial cells of the BBB are connected by Tight Junctions (TJ) [1], so that no fenestration between the cells exists. The TJ close the intracellular space between the endothelial cells and block the free diffusion of water-soluble polar substances. Therefore, these cells create a high transendothelial electrical resistance (TER) which yields *in vivo* values up to 2000 Ωcm^2^ [2], [3]. In addition, the brain capillary endothelial cells possess an increased number of mitochondria resulting in an increased metabolic activity. The brain capillary endothelial cells are surrounded by astrocytes, microglial cells, pericytes and nerve ends. They play an essential part in the maintenance of the BBB characteristics [4]. The BBB is involved in the regulation of the constancy of the internal environment of the brain and maintains an essential brain homeostasis. Only lipophilic and small hydrophobic molecules can cross the BBB by diffusion. However, many molecules falling into this category are not transported as they are substrates for the very efficient efflux transporters such as Pgp. Nevertheless, for some large molecules, peptides and proteins receptor-mediated specific transport systems do exist [5], [6]. As a result of its properties, the BBB enables a protection of the brain from the peripheral circulation and toxic substances but restricts the transport of many therapeutically important drugs from the blood into the brain [7], including anticancer drugs, Alzheimer disease drugs, antibiotics, and a wide variety of central nervous system (CNS)-active drugs. Because the BBB represents such an insurmountable obstacle for most drugs an effective treatment of many brain diseases is difficult or not possible. Therefore, a number of different strategies have been employed during the past years to overcome this barrier. These strategies included the osmotic opening of the tight junctions, the direct surgical administration of drugs into the brain, and the use of prodrugs or carrier systems like antibodies or liposomes [6], [7], [8], [9]. Later on, the use of nanotechnology came into play [10], [11], [12] and not only liposomes but also solid lipid nanoparticles or different polymeric nanoparticles [11] have successfully been used for the transport of drugs across the BBB. Thus, it was possible to transport an increasing number of nanoparticle-bound drugs including doxorubicin [13], [14], [15], dalargin [11], [16], [17], loperamide [18], [19], [20], and others [17], [21], [22] with different chemical properties and therapeutic effects over the BBB. Moreover, these nanoparticles have not only enhanced the transport of the drug into the brain but also protected the active agents from enzymatic degradation and were able to reduce side effects [23].

Some earlier work indicated that the binding of certain apolipoproteins to the nanoparticles provides an optimal tool to transport drugs over the BBB [19], [24]. It could be shown, for example, by means of two-dimensional polyacrylamid gel electrophoresis that after injection into the blood stream, apolipoprotein E (ApoE) was adsorbed onto the surface of polysorbate 80-coated nanoparticles, which then could enter the BBB [25], [26]. Further studies verified a clear correlation between the ApoE adsorption and the BBB passage. For instance, after coating with polysorbate 80 and/or adsorption of apolipoprotein E or B poly(butyl cyanoacrylate) nanoparticles were able to cross the BBB *in vivo* and thus transported bound dalargin or loperamide over this barrier [11], [16], [27]. The adsorption of other apolipoproteins except apolipoprotein A-I (ApoA-I) could not enable a pharmacological effect of these substances [27], [28]. Therefore, it was hypothesized that these nanoparticles resemble endogenously circulating lipoproteins [27], [29], [30] and are taken up by a receptor-mediated pathway by the brain endothelial cells which express the respective receptors [6], [22], [24], [27], [29], [30], [31]. The fact that nanoparticles made of human serum albumin with adsorbed or covalently bound ApoE or ApoA-I can transport drugs over the BBB [19], [27], [32], corroborated this assumption. Our other studies showed a specific binding and uptake of ApoE- or ApoA-I-modified human serum albumin nanoparticles on endothelial cells and an entrance into the CNS by transcytosis and a delivery to neurons [28], [33].

However, up to now the exact mechanism of this nanoparticle drug transport over the BBB was not fully known and the involved receptor not identified. The present study aimed at the elucidation of this uptake mechanism and the identification of this receptor and was able to show in *in vitro* experiments the involvement of a member of the low density lipoprotein receptor (LDLR) family, namely the low density lipoprotein receptor related protein (LRP1).

## Materials and Methods

### Nanoparticle preparation and characterization

#### Chemicals and Reagents

Human serum albumin (HSA, fraction V, purity 96–99%, 65000 Da) as well as glutaraldehyde 25% solution was purchased from Sigma-Aldrich (Schnelldorf, Germany). 2-Iminothiolane HCL (Traut's reagent) and D-Salt™ Dextran Desalting columns were obtained from Pierce (Rockford, USA). Recombinant apolipoprotein E3 (342000 Da) was produced as described by Vogel et al. [34]. The crosslinker Malhex-NH-PEG-COOSu (4800 Da) was purchased from RAPP Polymere GmbH (Tübingen, Germany) while the M-SPA-5000 PEGylating reagent (5356 Da) was bought from Nektar (Huntsville, USA). All other reagents and chemicals were purchased from Merck (Darmstadt, Germany) in analytical grade.

#### Preparation of the HSA nanoparticles

Unmodified human serum albumin (HSA) nanoparticles were produced using a desolvation technique previously described by Langer et al. [35] and Weber et al. [36], [37]. For this purpose, 200 mg of HSA were dissolved in 2 ml of a 10 mM NaCl solution. The desolvation with 8 ml of ethanol 96% (drop wise addition with a rate of 1 ml/min) was performed at a pH value of 8.0 under constant stirring to form the nanoparticles. The particles were then crosslinked with 200% glutaraldehyde (235 µl of an 8% solution) to stabilize the colloid. Purification of the particles was achieved by threefold centrifugation (8 min at 16100 g) and redispersion in ultra-pure water.

Apolipoprotein E3 was attached to the surface of unmodified HSA particles via a bifunctional Mal-PEG-NHS crosslinker according to Michaelis et al. [38]. The poly (ethylene glycol) crosslinker reacts with an amino group on the particle's surface as well as a thiol group introduced into the ApoE thus covalently linking the two reaction partners. The thiolation of the ApoE was achieved by incubating 1 mg of the apolipoprotein with a 50-fold molar excess of 2-Iminothiolane HCL (Traut's reagent) in phosphate buffer at room temperature for 2 hours. The thiolated ApoE was then purified using a D-Salt™ Dextran Desalting column and incubated with the crosslinker-activated nanoparticles for 12 hours. The resulting ApoE-modified nanoparticles were purified by threefold centrifugation and redispersion in ultra-pure water.

As a control, HSA nanoparticles with poly (ethylene glycol) (PEG) chains on their surface were prepared by linking mPEG-SPA-5000 to the particle surface of unmodified HSA nanoparticles. For this purpose 20 mg of HSA nanoparticles were incubated with a 50-fold molar excess of mPEG-SPA-5000 (82.4 mg) in phosphate buffer (pH 8.0) under constant shaking at room temperature for one hour. The resulting PEGylated particles were purified by threefold centrifugation and redispersion in ultra-pure water.

#### Nanoparticle Characterization

The nanoparticle preparations were characterized concerning their size, polydispersity and zeta potential using photon correlation spectroscopy (Zetasizer 3000 HS_A_, Malvern, Germany). The dynamic light scattering measurements were performed in aqueous suspension at 25°C and a measuring angle of 90°. The concentration of the nanoparticle suspensions was determined by microgravimetry and set to 10 mg/ml by diluting the suspension with ultra-pure water.

### Cell culture

For the *in vitro* cell culture studies the mouse brain endothelioma cell line bEnd3 (LGC Promochem, Wesel, Germany) was used. The cells were cultured at 37°C and 5% CO_2_ in DMEM high glucose medium, supplemented with 10% fetal calf serum.

### Cellular binding of the nanoparticles

For the cellular binding studies the bEnd3 cells were cultivated on collagen IV-coated (Sigma-Aldrich, Steinheim, Germany) 24-well plates until a post confluent monolayer had grown. Then, the cells were incubated with 0.1 mg/ml of the different nanoparticulate formulations for 4 h (an established incubation time [33]) at 4°C and 37°C, respectively. Afterwards, the cells were washed twice with PBS (Invitrogen, Karlsruhe, Germany), then trypsinized and harvested. After washing with PBS and fixing with FACS-Fix (10 g/l PFA and 8.5 g/l NaCl in PBS, pH 7.4) flow cytometry (FACS) analysis was performed with 10,000 cells/sample, using FACSCalibur and CellQuest Pro software (Becton Dickinson, Heidelberg, Germany). Due to a green autofluorescence of these nanoparticles at 488/520 nm this FACS analysis was possible.

### Cellular uptake and intracellular distribution of the nanoparticles

Cellular uptake and intracellular distribution of the nanoparticles were studied by confocal laser scanning microscopy (CLSM). bEnd3 cells were cultured on collagen IV-coated glass slides and treated with the different nanoparticle formulations for 4 h at 37°C. After this incubation the cells were washed twice with serum-free medium and the cytosol was stained with CellTracker™ Red CMTPX (Invitrogen, Karlsruhe, Germany) as described in the manufacturer instructions manual. Cells were fixed with 0.5–1% PFA for 5–10 min. After fixation the cells were embedded in Vectashield HardSet Mounting Medium containing DAPI for cell nuclei staining. The CLSM study was performed with an Axiovert 200 M microscope with a 510 NLO Meta device (Zeiss, Jena, Germany), Ti:Sa femtosecond or an argon ion laser and the LSM Image Examiner software. The green autofluorescence of the nanoparticles at 488/520 nm was used here.

### Uptake mechanism study

#### Determination of the receptor state of the brain endothelial cells

For the determination of the receptor state flow cytometry analysis was also used. The bEnd3 cells were cultivated on collagen IV-coated well plates until a post confluent monolayer had grown. Then, the cells were permeabilized, fixed and blocked with 5% goat serum (20 min, 4°C) before they were incubated with the primary antibodies against the ApoE receptor (ApoER) (Acris Antibodies GmbH, Herford, Germany), Megalin (abcam, Cambridge, UK), LDLR (abcam, Cambridge, UK) or LRP1 (Pietrzik et al. 2002 [39]) for 30 min at 4°C. The antibodies were used in concentrations according to the manufactures' instructions and more diluted, respectively. Afterwards, the cells were washed with PBS and incubated with the corresponding secondary antibody (Invitrogen, Karlsruhe, Germany) for 30 min at 4°C. After washing with PBS and fixing with FACS-Fix (10 g/l PFA and 8.5 g/l NaCl in PBS, pH 7.4) flow cytometry (FACS) analysis was performed with 10,000 cells/sample, using FACSCalibur and CellQuest Pro software. As an antibody control the cells were incubated only with the secondary antibody and also analyzed.

#### GST fusion proteins purification

LRP1 ligand binding domains and RAP were subcloned into the pGEX-4T vector (Amersham Pharmacia Biotech, Piscataway, New Jersey, USA). Plasmids were transformed into Escherichia coli BL21 and protein expression was induced by 1 mM IPTG (Roth, Karlsruhe, Germany) for 4 h. After bacterial lysis in 3% sarkosyl buffer cell debris were spun down at 14,000 rpm. The protein was pulled down using glutathione-Sepharose beads (Amersham Biosciences, Piscataway, New Jersey, USA) and eluted with a 10 mM glutathione in 50 mM Tris solution pH 7.4.

#### Co-incubation experiments

The co-incubation experiments were also performed by flow cytometry analysis. The bEnd3 cells were cultivated on collagen IV-coated 24-well plates until a post confluent monolayer had grown. Then, the cells were either incubated with 0.1 mg/ml of the different nanoparticulate formulations for 4 h at 37°C or co-incubated with 0.1 mg/ml of the different nanoparticulate formulations and 1 mg/ml low density lipoprotein (LDL) (Calbiochem/VWR, Darmstadt, Germany), 500 nM RAP+1 mg/ml LDL or 0.125 mg/ml LRP1 Dom II and 0.125 LRP1 Dom IV, respectively. Afterwards, the cells were washed twice with PBS, then trypsinized and harvested. After washing with PBS and fixing with FACS-Fix (10 g/l PFA and 8.5 g/l NaCl in PBS, pH 7.4) flow cytometry (FACS) analysis was performed with 10,000 cells/sample, using FACSCalibur and CellQuest Pro software. Due to a green autofluorescence of these nanoparticles at 488/520 nm this FACS analysis was possible.

## Results

### Nanoparticle preparation and characterization

The human serum albumin-based nanoparticles were prepared by a well-known desolvation technique previously described by Langer et al. [35] and Weber et al. [36], [37]. As summarized in Table 1, the size of the ApoE-modified nanoparticles was 197.8±4.8 nm and the size of the PEGylated nanoparticles was 186.7±0.4 nm. The zeta potential of the nanoparticulate formulations was between −26.6±7.5 and −42.5±6.3 and the polydispersity index of all preparations was smaller than 0.1 demonstrating that monodisperse nanoparticle formulations have been prepared. The high surface charge of both nanoparticle preparations guaranties the colloidal stability of both systems. The apolipoprotein molecules covalently bound on the particle surface produced a higher zeta potential of the ApoE-modified nanoparticles in comparison to the PEGylated nanoparticles due to the higher number of chargeable groups.

**Table 1:** Nanoparticle characterization.

	NP-PEG	NP-ApoE
Particle diameter [nm]	186.7±0.4	197.8±4.8
Polydispersity	0.03±0.03	0.03±0.03
Zeta potential [mV]	−26.6±7.5	−42.5±6.3

### Cellular binding of the nanoparticles

**Figure 1:**
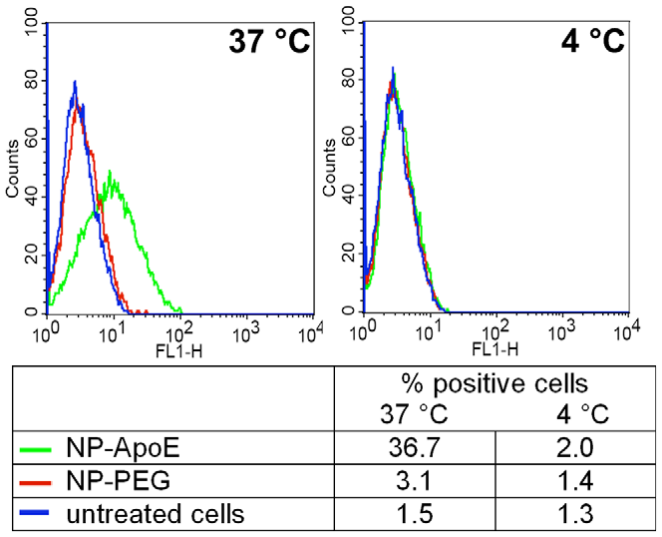
Specific cellular binding of the ApoE-modified nanoparticles studied by flow cytometry. bEnd3 cells were incubated with ApoE-modified nanoparticles (NP-ApoE) or control nanoparticles without ApoE modification (NP-PEG) for 4 h at 37°C and 4°C, respectively. Flow cytometry analysis was performed to quantify their cellular binding. The data are shown as histograms of the FL1-H-channel (autofluorescence of the nanoparticles) as well as in the table with the analysis of the Y mean fluorescence and the percentage of positive cells. Green: NP-ApoE, red: NP-PEG, blue: untreated control.

For the clarification of the uptake mechanism of ApoE-modified nanoparticles, first of all, the cellular binding of the nanoparticles had to be proven by flow cytometry analysis. Therefore, the mouse brain endothelioma cells bEnd3 were incubated either with the specific ApoE-modified nanoparticles (NP-ApoE) or the unspecific PEGylated control nanoparticles (NP-PEG) for 4 h at 37°C or at 4°C. The histograms of both incubation temperatures are compared in Figure 1. At 37°C a specific cellular binding of the ApoE-modified nanoparticles could be detected by 36.7% of positive cells. In comparison, the cellular binding of the control nanoparticles was really low with 3.1% of positive cells demonstrating a marginal unspecific binding. In contrast, at an incubation temperature of 4°C almost no cellular binding of the unspecific and the specific nanoparticles could be observed (less than 2% of positive cells).

### Cellular uptake and subcellular distribution of the nanoparticles

**Figure 2:**
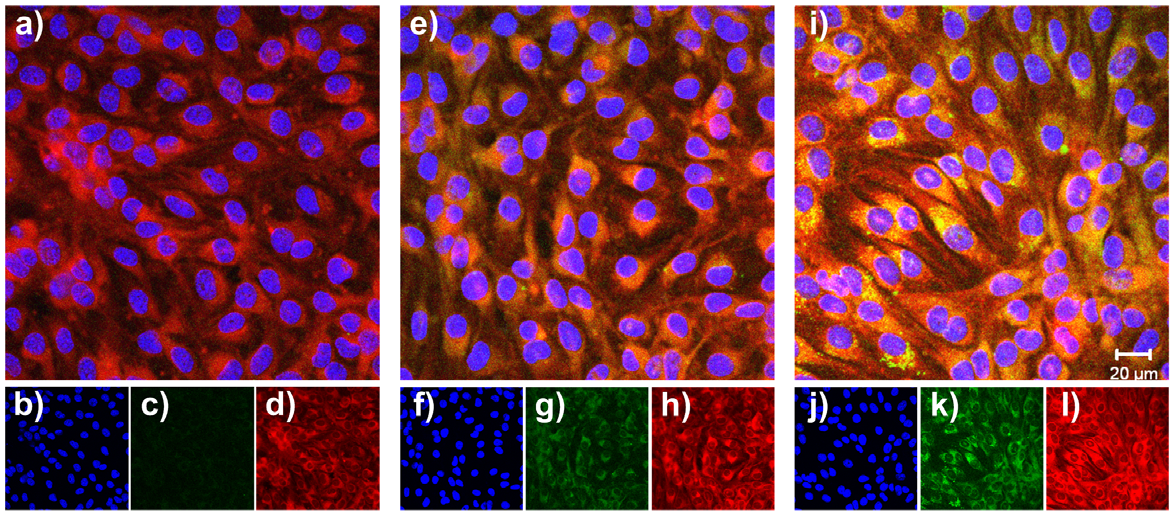
Cellular uptake and intracellular distribution of the nanoparticles studied by CLSM: split of the fluorescence channels. bEnd3 cells were incubated for 4 h with 0.1 mg/ml of the different nanoparticulate formulations at 37°C. The green autofluorescence of the nanoparticles was used for detection. The cytosol was stained in red with CellTracker™ Red CMTPX, and the nucleus was stained in blue with DAPI. Pictures were taken within inner sections of the cells. Untreated control cells: a) overlay of all fluorescence channels, b) display of the blue nucleus channel, c) display of the green nanoparticle channel, d) display of the red cytosol channel. Cells with the unspecific control NP-PEG: e) overlay of all fluorescence channels, f) display of the blue nucleus channel, g) display of the green nanoparticle channel, h) display of the red cytosol channel. Cells with the specific NP-ApoE: i) overlay of all fluorescence channels, j) display of the blue nucleus channel, k) display of the green nanoparticle channel, l) display of the red cytosol channel.

For studies concerning the cellular uptake and intracellular distribution of the nanoparticles the confocal laser scanning microscopy (CLSM) was used. The bEnd3 cells were incubated as well with the unspecific PEGylated control nanoparticles as with the specific ApoE-modified nanoparticles for 4 h at 37°C. In case of the incubation with the specific ApoE-modified nanoparticles a clear intracellular uptake and accumulation could be observed in contrast to the unspecific control nanoparticles (Figure 2).

These findings argued for a receptor-mediated uptake of the specific ApoE-modified nanoparticles. Thus, for clarification of the uptake mechanism and receptor identification, typical receptors located at the BBB were investigated.

### Uptake mechanism study

#### Determination of the receptor state of the brain endothelial cells

For the determination of the receptor state, the bEnd3 cells were incubated with antibodies against the low density lipoprotein receptor related protein (LRP1), the low density lipoprotein receptor (LDLR), the ApoE-receptor (ApoER) and Megalin in different concentrations. The bEnd3 cells expressed all these receptors as indicated in Table 2. With nearly 100% of positive cells the receptors Megalin, LRP1 and LDLR were clearly more present than the ApoER with 21.1% of positive cells.

**Table 2:** Determination of the receptor state of the bEnd3 cells.

receptor	dilution of the primary antibody	% positive cells
**Megalin**	C	1.6
	1∶100	81.7
	1∶50	96.2
	1∶10	99.3
**ApoER**	C	1.6
	1∶100	3.2
	1∶20	10.0
	1∶10	21.1
**LRP1**	C	1.5
	1∶1000	95.3
	1∶500	98.0
**LDLR**	C	1.5
	1∶70	97.9

C: control only with the secondary antibody.

The involvement of a certain receptor of the LDLR family then was specifically investigated in different co-incubation experiments.

#### Co-incubation experiments

In a first co-incubation experiment the bEnd3 cells were incubated with the different nanoparticulate formulations together with the low density lipoprotein (LDL). Cells incubated solely with nanoparticles were used as reference. At first, the specific targeting of the ApoE-modified nanoparticles in contrast to the PEGylated control nanoparticles could be observed (Table 3) corroborating the results obtained in the binding study. The co-incubation of the nanoparticles with LDL and the specific ApoE-modified nanoparticles led to an increased number of positive cells with an enhancement of 31.2% to 42.6% of positive cells whereas the co-incubation with the unspecific nanoparticles showed only a marginal enhancement of 3.3% to 4.5% of positive cells (Table 3).

**Table 3:** Co-incubation of bEnd3 cells with the different nanoparticulate formulations and LDL.

	NP incubation [% positive cells]	NP+LDL co-incubation [% positive cells]
NP-ApoE	31.2	42.6
NP-PEG	3.3	4.5
untreated control	1.7	1.1*

*: cells without NP incubation, with LDL.

One representative experiment out n>3 is shown.

Additional evidence for LDLR family member involvement in ApoE nanoparticle uptake was generated by co-incubation experiment using the receptor-associated protein (RAP). RAP blocks all binding sites of most LDL receptor family members. Therefore, the application of RAP to the tissue culture medium led to a clear reduction of the number of positive cells (25.1% to 7.1% of positive cells) binding ApoE-modified nanoparticles. In contrast, the binding of the unspecific nanoparticles to the bEnd3 cells increased slightly from 3.2% to 8.4% of positive cells (Table 4).

**Table 4:** Co-incubation of bEnd3 cells with the different nanoparticulate formulations and LDL+RAP.

	NP incubation [% positive cells]	NP+LDL+RAP co-incubation [% positive cells]
NP-ApoE	25.0	7.1
NP-PEG	3.2	8.4
untreated control	1.1	2.0*

*: cells without NP incubation, with LDL+RAP;

One representative experiment out n>3 is shown.

In a second co-incubation experiment the bEnd3 cells were incubated with the different nanoparticles and purified soluble fragments of the LRP1. These fragments express the binding domains II and IV of LRP1, (LRP1 Dom II and LRP1 Dom IV) and were used to unravel which of LRP1 domain might be involved in the specific ApoE-mediated nanoparticle binding. After exogenous addition to the culture medium both fragments induced an inhibition of the specific nanoparticle binding to the bEnd3 cells compared to the control experiment with 16.1% of positive cells (Table 5). However the incubation with the LRP1 Dom IV caused a larger inhibitory effect (0.5% of positive cells) than the LRP1 Dom II (10.4% of positive cells) indicating that this domain might have a stronger effect on ApoE nanoparticle binding.

**Table 5:** Co-incubation of bEnd3 cells with the different nanoparticulate formulations and LRP1 Dom II and LRP1 Dom IV, respectively.

	NP incubation [% positive cells]	NP+LRP1 Dom II co-incubation [% positive cells]	NP+LRP1 Dom IV co-incubation [% positive cells]
NP-ApoE	16.1	10.4	0.5
NP-PEG	1.4	2.2	1.6

One representative experiment out n = 3 is shown.

## Discussion

In our former mouse *in vivo* experiments ApoE-modified HSA-based nanoparticles could be recovered in different brain regions and neurons by transmission electron microscopy (TEM) images already 15 minutes after their intravenous injection [33]. However, the tight junctions in the brain regions remained intact, indicating a specific endocytotic uptake of the ApoE-modified nanoparticles at the luminal site of the endothelial cells and further transcytosis into the brain at the abluminal site. In contrast, the PEGylated nanoparticles could be detected only in minor amounts in the endothelial cells and were not found at all in the residual brain regions even 30 minutes after injection. This fact confirms the unspecificity of the PEGylated nanoparticles. Corresponding *in vitro* TEM investigations with the murine brain endothelioma cell line bEnd3 and murine *in vivo* TEM investigations confirmed the results that the nanoparticles were intracellular endocytosed by formed pits. However, up to now the exact uptake mechanism of these nanoparticles were not fully understood. Therefore, in order to elucidate the nanoparticle transport mechanism over the BBB ApoE-modified HSA-based nanoparticles and corresponding PEGylated control nanoparticles were manufactured according to an established process [35], [36], [37] and were tested on the former established murine brain endothelioma cell line bEnd3. These cells are suitable for studies addressing blood-brain barrier transport mechanism according to Omidi et al. [40], who characterized the bEnd3 cell line. The synthesized particles were in a size range of about 200 nm and possessed a monodisperse particle size distribution qualifying these particles for mechanism studies. Furthermore, as already shown in our former publication [33], these nanoparticles are not cytotoxic in the tested concentration range.

First of all, clearly a specific cellular binding on the mouse brain endothelioma cells bEnd3 was confirmed for the ApoE-modified nanoparticles by flow cytometry, although at this stage the corresponding receptor on the cellular side was not known. In contrast, the PEGylated control nanoparticles showed only a marginal cellular binding, which can be considered to be unspecific. This experiment was performed at 37°C, a temperature which is generally important for the maintenance of cellular functions. However, when this experiment was conducted at 4°C no specific cellular binding could be detected and the unspecific binding part was reduced to the same level like the untreated cells. At this temperature all active, energy consumptive transport processes are stopped. These findings argued for an active endocytotic uptake mechanism of the nanoparticles into the cells and imply a receptor-mediated transcellular uptake pathway of the nanoparticles over the endothelial cells into the brain.

Furthermore, for the analysis of the cellular uptake and subcellular distribution of the nanoparticulate formulations the confocal laser scanning microscopy was used and a significant intracellular accumulation could be observed. This result confirms our former TEM investigations [33] where the intracellular nanoparticle endocytosis by formed pits was shown. All these findings supported former assumptions [27], [29], [30], [32], that the nanoparticulate transport of drugs over the BBB is a receptor-mediated endocytotic process involving the selective binding between ApoE and the respective receptor belonging to the LDL receptor family. This assumption was further confirmed by experiments with nanoparticles which were modified with an ApoE-related nonsense sequence. These nanoparticles were unable to transport bound drugs over the BBB [32].

Even though the incubation times and nanoparticle concentrations are different *in vivo* and *in vitro*, it is realistic to assume that the same receptors are involved *in vivo* and *in vitro*. Therefore, in the scope of the present study further specific experiments concerning the nanoparticulate transport mechanism of the ApoE-modified HSA-based nanoparticles over the BBB were performed in *in vitro* cell culture systems. As shown in this investigation the bEnd3 cells express the LRP1, LDLR, Megalin and at lower level the ApoER. Therefore, these cells were used for the transport mechanism study. Moreover this receptor expression pattern of the bEnd3 cells indicated an involvement of the LDL receptor family by the specific nanoparticle uptake and therefore the LRP1, a member of the LDL receptor family [41], was especially investigated.

The co-incubation of the bEnd3 cells with the different nanoparticulate formulations and LDL led to an enhanced uptake of the specific ApoE-modified nanoparticles. The presence of the LDL could induce a conformation change of the ApoE structure. This may lead to an enhanced binding capacity of the ApoE to the receptor since it is known that lipid-free ApoE does not bind to the LDL receptor with high affinity and for the high affinity binding an association with lipids such as phospholipids or lipoproteins is required. [42], [43], [44]. Furthermore, the lipid composition has an influence on the conformation of ApoE and therefore on the receptor affinity [45].

Consequently, the enhanced specific uptake effect induced by LDL was reversed by co-incubation with the receptor associated protein RAP. In contrast, the cellular binding of the unspecific nanoparticles was slightly enhanced. RAP is a protein that blocks all binding sites on most receptors of the LDLR family and acts a chaperon for LRP1 which is a member of this receptor group. It enables the correct intracellular folding of the LRP1 through binding on the ligand binding sites and prevents an early ligand-receptor interaction [46], [47]. In the *in vitro* culture, administration of purified RAP blocks binding of LRLR ligands to receptors on cellular surface. Consequently, the binding of ApoE attached to the surface of the nanoparticles on the extracellular receptor in the present experiments was also inhibited, demonstrating the participation of the LDL receptor family in the nanoparticles' uptake. With this experiment the specific nanoparticle binding on cellular site could be inhibited.

Taking especially the LRP1 into account, further experiments then focused on the question which binding domain of the LRP1 is involved in the specific ApoE-mediated nanoparticle binding on the cells. Therefore, the nanoparticles were co-incubated with soluble fragments of the LRP1, which contained the main LRP1 binding domains II and IV, respectively [48]. In principle, with both fragments an inhibition of the specific nanoparticle binding could be induced in the present study whereby the incubation with LRP1 Dom IV showed the stronger effect. It is a well-known fact that both binding domains have been shown to bind numerous LRP1 ligands [48]. With this experiment the specific nanoparticle binding on nanoparticulate site could be inhibited. This experiment provides first evidence that soluble LRP1 domains can sequester ApoE-modified nanoparticles away from the cellular bound receptor. Indicating that LRP1, which shows one of the highest expression of all LDL receptors in bEnd3 cells, might act as a transporter for ApoE-modified nanoparticles.

Within this experiment also a slight enhancement of the unspecific binding rate could be verified. In general, it seems that the cells enhance the unspecific nanoparticle uptake if the specific uptake pathway is inhibited.

This data clearly demonstrates the participation of LDL receptor family members, especially LRP1, on the specific ApoE-mediated nanoparticle uptake on brain endothelial cells.

A participation of apolipoproteins in the transport of nanoparticle-bound drugs over the BBB was already considered in earlier experiments with polysorbate 80-coated poly(butyl cyanoacrylate) (PBCA) nanoparticles. It was anticipated that these particles after injection into the blood stream adsorb certain apolipoproteins on their surface and as a result mimic circulating lipoproteins [27], [29], [30] and thus could interact with the apolipoprotein receptors of the BBB followed by their endo- and transcytosis. The present studies confirmed these assumptions in that similar uptake processes are taking place with the surfactant-coated and apolipoprotein-adsorbed as with particles with covalently bound apolipoprotein that was used here. In addition, the selective neuronal uptake in *in vitro* primary cells was previously shown for the polysorbate 80-coated PBCA nanoparticles, which could be inhibited by an LDLR antibody [49]. Moreover, the employment of apolipoprotein fractions containing only a binding sequence appears to be sufficient [50], [51], [52], [53].

Due to the understanding of the uptake and transport mechanism of nanoparticulate formulations into the brain a rational design of appropriate nanoparticles and the tailoring of very specific and effective carriers seems to be feasible.
